# 2571. Posaconazole DRT in Children; Time to Bring Out the Pill Crusher?

**DOI:** 10.1093/ofid/ofad500.2188

**Published:** 2023-11-27

**Authors:** Heather L Weerdenburg, Amanda Gwee, Gabrielle Haeusler, Joshua Osowicki, Alison Boast

**Affiliations:** Royal Children's Hospital Melbourne, Melbourne, Victoria, Australia; The Royal Children's Hospital, Melbourne, Victoria, Parkville, Victoria, Australia; Royal Children's Hospital Melbourne, Melbourne, Victoria, Australia; Murdoch Children's Research Institute & Royal Children's Hospital Melbourne, Melbourne, Victoria, Australia; Royal Children's Hospital Melbourne, Melbourne, Victoria, Australia

## Abstract

**Background:**

Posaconazole, a broad-spectrum triazole antifungal, is licenced for prophylaxis and treatment of invasive fungal infections (IFI). Young children who are unable to swallow tablets or those with enteral tubes are routinely prescribed oral suspension. However, this formulation has poor bioavailability and despite appropriate dosing, often results in subtherapeutic concentrations. In contrast, the delayed release tablet (DRT) has excellent bioavailability irrespective of gastric pH resulting in reliable absorption and attainment of therapeutic exposure. Several adult case series have reported the achievement of therapeutic posaconazole concentrations by crushing DRT. We investigated the attainment of therapeutic posaconazole concentrations with crushed DRT.

**Methods:**

Case series of children receiving posaconazole DRT via enteral tube between September 2022 to April 2023. Each 100mg DRT was crushed in ∼10ml of sterile water followed by a flush of ∼10ml - 20mL to avoid blocking the tube. Tablets were not given with food. Therapeutic trough serum concentrations were defined as ≥ 0.7mg/L (prophylaxis) and ≥ 1mg/L (treatment).

**Results:**

Four children, aged 4 to 14 years, received crushed posaconazole DRT (treatment; n=3, prophylaxis; n=1). The median initial dose was 10 mg/kg/d (range 5 -11). All children achieved target concentrations, however, two children receiving posaconazole for treatment of IFI required a dose increase prior to achieving therapeutic concentrations. No toxicities were reported. The switch to posaconazole DRT facilitated earlier hospital discharge for 2 patients.

**Figure:**
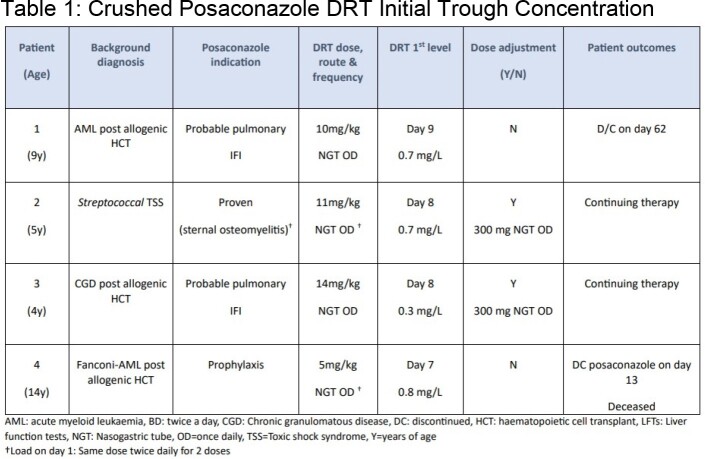

Posaconazole Trough Levels For IFI Treatment

**Figure:**
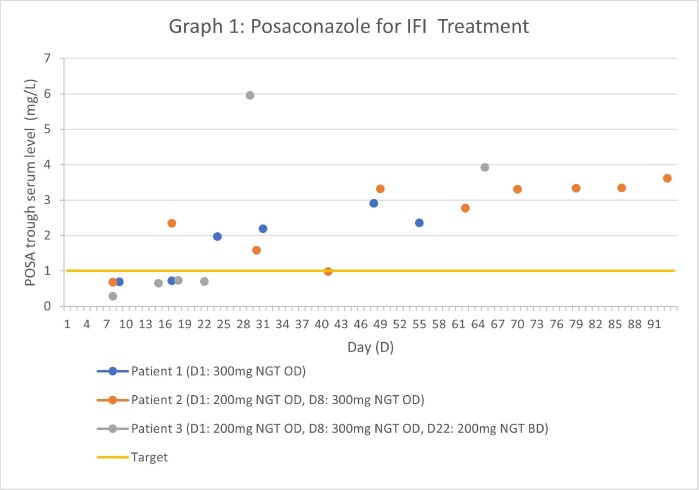

**Conclusion:**

Our case series demonstrates that therapeutic concentrations can be achieved when crushed poscaonzole DRT is administered via enteral tube. This formulation allowed the discontinuation of IV formulations, facilitating earlier patient discharge.

**Disclosures:**

**All Authors**: No reported disclosures

